# A Pulmonary Zebra: Adult Primary Pulmonary Synovial Sarcoma

**DOI:** 10.1155/2022/8649540

**Published:** 2022-04-16

**Authors:** Sheffield Sandra, Nwachukwu Chidi, Ashby Tracy

**Affiliations:** Department of Medicine, UF College of Medicine – Jacksonville, 653-1 West 8th Street, L20 4th Floor, LRC Jacksonville, FL 32209, USA

## Abstract

Primary pulmonary synovial sarcoma (PPSS) is an extremely rare tumor, with approximately 50 cases being reported in the English literature (Golota et al., 2018). Difficulties are often encountered in the diagnosis of PPSS as it can be confused with other spindle or round cell tumors, such as fibrosarcoma, hemangiopericytoma, mesothelioma, sarcomatoid carcinoma, or metastatic sarcomas. PPSS was first described by Zeren et al. in 1995. We present a case of PPSS in a 41-year-old woman, who complained of progressive dyspnea and left-sided chest pain.

## 1. Introduction

Sarcomas are a diverse group of malignant tumors. Sarcomas predominantly arise from the embryonic mesoderm [1]. More than 100 sarcoma subtypes have been identified with distinctive pathology, molecular characteristics, and clinical presentations. Sarcomas represent <1% of adult cancers and account for approximately 21% of pediatric malignancies [2]. There are two main categories of sarcomas: (1) tumors arising from the soft tissues (e.g., muscle, tendons, and blood vessels) and (2) those arising from the bone (e.g., osteosarcoma and Ewing's sarcoma). Approximately 80% arise from soft tissue and 20% originate from the bone. The World Health Organization has defined approximately 50 soft tissue sarcoma histologic subtypes [1]. Soft tissue sarcomas include angiosarcoma, liposarcoma, leiomyosarcoma, neurofibrosarcoma, schwannoma, Kaposi's sarcoma, rhabdomyosarcoma, vascular sarcoma, fibrosarcoma, mesenchymomas, myxofibrosarcoma, gastrointestinal stromal tumor, and synovial sarcomas.

Synovial sarcomas (SS) are one of the most common soft tissue tumors in adolescents and young adults, and it accounts for approximately 5–10% of all soft tissue sarcomas [3]. About one-third of cases occur in the first two decades of life with an average age of 30 years at diagnosis. The origin of synovial sarcoma is unclear. However, SS is associated with a recurrent reciprocal translocation between chromosomes X and 18, t(X; 18) (p11.2; q11.2), which leads to the expression of several different SS18:SSX fusion proteins. The fusion proteins SYT-SSX1 and SYT-SSX2 are believed to function as aberrant transcriptional regulators, resulting in either activation of protooncogenes or inhibition of tumor suppressor genes [4].

Primary SS is most common in the large joint of the extremities. However, primary SS have been documented in most human tissues and organs including the neck, brain, prostate, tongue, kidney, larynx, mediastinum, esophagus, heart, abdomen wall, small intestine, mesentery, vessels, retroperitoneum, and lungs [5]. PPSS can originate from vessels, pulmonary stroma, and mesenchymal elements of the bronchial wall. PPSS accounts for 0.1% to 0.5% of all primary lung malignancies [6]. Most of the available literature concerning PPSS is limited to case reports and case series [7].

## 2. Case Report

A 41-year-old female of Indian descent with no significant past medical history presented to the emergency room with a 2-month history of progressive dyspnea on exertion and sharp, pleuritic, substernal chest pain. She also endorsed anxiety and chest palpitations, denied edema, nausea, vomiting, fevers, and chills, and has no history of tobacco, alcohol, or illicit drug use.

On physical examination, vital signs revealed a heart rate of 126 beats per minute and a respiratory rate of 32 breaths per minute. She was pale with decreased respiratory movement on the left supraclavicular and infraclavicular areas. There was no cervical lymphadenopathy or clubbing.

**Figure 1:**
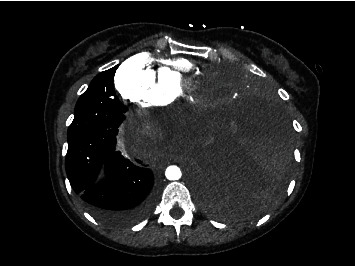
CT axial view arterial phase postcontrast large 17.9 × 17.6 × 18.3 cm heterogeneous mass centered within the left hemithorax, resulting in rightward mediastinal shift. An incidental right pleural effusion is present.

**Figure 2:**
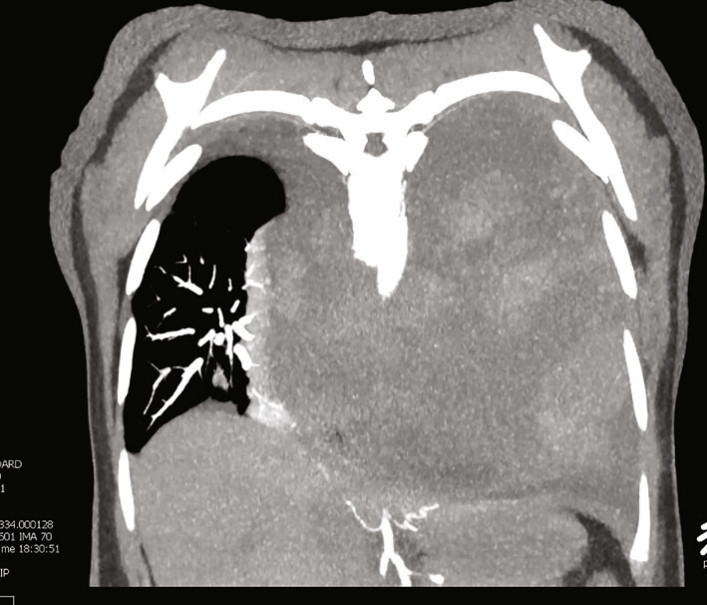
CT chest coronal MIP (maximum intensity projection) rightward mediastinal shift and partial right lung collapse secondary to left hemithoracic mass effect.

**Figure 3:**
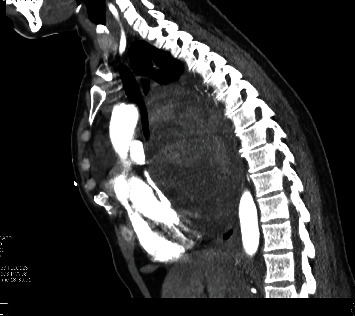
CT sagittal projection centered along the midline depicted anterior displacement of mediastinal structures secondary to larger left hemithoracic mass, which crosses the midline.

Initial blood gas analysis was pH 7.2/CO2 47.9/HCO3 28/PO2 of 70. An electrocardiogram revealed sinus tachycardia. Cardiac biomarkers were negative. Complete hemogram revealed normocytic anemia with a hemoglobin level of 11.5 g/dl (reference [8]-16 g/dl), and a complete metabolic panel revealed a bicarb level of 35 (reference 21-29 mmol/l). A chest radiograph revealed opacification of the left hemithorax with a mass effect on the mediastinal structures. A chest computer tomography (CT) revealed a large mixed attenuated mass involving the left chest and mediastinum measuring 17.9 × 17.6 × 18.3 cm in dimension (Figures 1, 2, and 3). Furthermore, a video-assisted thoracoscopic biopsy from the lung mass showed an infiltrating spindle cell neoplasm. The immunohistochemistry of the tissue sample revealed cells expressing epithelial membrane antigen (EMA), CD99, BCL-2, and calponin. The cells were immune-negative for cytokeratin and S-100.

## 3. Case Discussion

PPSS is a rare and highly aggressive type of SS, usually occurring in young adults. It generally presents as a large, circumscribed mass. Metastatic SS from extremities is common in the pulmonary parenchyma and pleura. Patients with PPSS often present with chest wall pain, cough, dyspnea, hemoptysis, and ipsilateral pleural effusions. There may be a slight male predilection, but there is no relation to smoking status [9]. Toxic substances and radiation have been noted as risks factors. Furthermore, reciprocal translocations between chromosomes X and 18 and infectious pathogens have been noted to have an impact on the origin of PPSS.

The diagnosis of PPSS requires clinical, pathological, and immunohistochemical investigations and radiological findings to exclude alternative primary lung tumors and metastatic sarcomas [9, 10]. Primary pulmonary synovial sarcomas are composed of two morphologically different cell types: (1) epithelial cells or (2) fibroblast-like spindle cells [11]. Histopathologically, there are four subtypes of PPSS, monophasic fibrous cell and biphasic; less common subtypes include monophasic epithelial and poorly differentiated (round cell) tumors [12]. Immunohistochemically, PPSS is positive for markers such as CD99, Bcl-2, vimentin, desmin, actin, cytokeratin, and EMA [2]. Based on radiologic imaging alone, manifestations of PPSS are indistinguishable from other common pulmonary and pleural neoplasms [6]. Typical CT imaging findings are large, heterogeneous tumors with internal necrosis. Radiologic findings are useful for delineating the extent of tumor involvement, determining the potential for surgical resection, and monitoring the effects of chemotherapy or radiation therapy. Our case was characterized by a well-defined heterogeneous enhancing mass of the left hemithorax with the presence of spindle cell sarcoma on histopathological examination and the expression of positive tumor markers for EMA, CD99, BCL-2, and calponin.

Currently, there is no standardized therapy for patients with PPSS. Treatment options include surgery, chemotherapy, radiation, and recently adoptive immunotherapy using tumor-infiltrating lymphocytes [13]. The preferred modality of treatment for PPSS is complete surgical resection. The standard surgical strategy of PPSS includes lobectomy and pneumonectomy. According to Golota et al., the treatment of choice for PPSS is anatomical resection of lung tissue with a tumor-free margin. Male patients and those with tumors larger than 5 cm have worse prognoses. Golota et al. also reported higher overall survival in patients who underwent nonanatomical resections versus those who underwent anatomical resections.

For unresectable tumors, chemotherapy, radiotherapy, and immunotherapy can be considered [3, 6, 12]. PPSS is considered relatively chemosensitive to chemotherapy agents such as ifosfamide, doxorubicin, and dacarbazine [14]. Robbins et al. tested the effectiveness of adoptive immunotherapy with genetically engineered lymphocytes that targeted the NY-ESO-1 antigen expressed in patients with advance synovial cell sarcomas, demonstrating that treatment with engineered lymphocytes mediates tumor regression in patients with metastatic synovial cell sarcomas [13]. The overall survival of patients with PPSS is poor, with a 5-year survival rate of 50%. Future studies are needed to identify ideal therapeutic agents for the treatment of PPSS.

## 4. Conclusion

PPSS is a very rare and aggressive tumor that should be included on the differential for lung and pleural masses. Due to poor prognosis, prompt diagnosis and treatment is imperative.

## Data Availability

The CT images used to support the findings noted on this case report were collected during the patient hospitalization. The data presented on this study have not been made available because the patient is deceased.
